# Effect of a low dose whey/guar preload on glycemic control in people with type 2 diabetes-a randomised controlled trial

**DOI:** 10.1186/1475-2891-13-103

**Published:** 2014-10-25

**Authors:** Peter M Clifton, Claire Galbraith, Leah Coles

**Affiliations:** University of South Australia, North Terrace, GPO Box 2471, Adelaide, SA 5001 Australia; Baker IDI Heart and Diabetes Institute, Prahan, VIC Australia

**Keywords:** Whey, Guar, Glycemic control, Type 2 diabetes

## Abstract

**Objective:**

Large preloads of protein and fat have been shown to lower glucose after a carbohydrate-rich meal in people with type 2 diabetes but add a considerable energy burden. Low calorie preloads [<5% of daily energy intake] have been tested in this study in people with prediabetes and with type 2 diabetes.

**Research design and methods:**

This was an unblinded randomised crossover study with two placebo days and two active treatment days. Glucose was measured for 3 hours with fingerprick samples as well as continuous glucose monitoring [CGMS]. Twenty-four subjects with pre-diabetes or moderately controlled type 2 diabetes [fasting glucose < 10 and HbA1c < 8.5%] were recruited. The preload contained 17 g whey protein plus 3 g lactose and 5 g guar, and 1 g flavour material [including sucralose] dissolved in 150 ml cold water or 150 ml cold water with no additives. The breakfast test meal consisted of 2 slices of bread, margarine and jam [3 slices for men] with the test drink 15 minutes beforehand.

**Results:**

Peak fingerprick glucose was reduced by 2.1 mmol/L at 45 min [p < 0.0001]. Average fingerprick glucose over 3 hours was reduced by 0.8 mmol/L [p = 0.0003]. There was no difference between those with diabetes or prediabetes or those on medication or not on medication.

**Conclusions:**

An 80 kcal whey protein/fibre preload can lower average glucose over 3 hours by 0.8 mmol/L. If used long term before at least two carbohydrate-rich meals/day this preload could lower HbA1c by up to 1%.

**Trial registration:**

The trial was registered with the Australian New Zealand Clinical Trials Registry number ACTRN12612001251819.

## Introduction

At least 50% of people with type 2 diabetes have poor glucose control despite drug treatment and are often faced with the prospect of the addition of another oral drug or insulin. Medical nutrition therapy involving lifestyle modification is recommended as the first line of management for this disease [1] but can also have significant effects on glucose control later in the disease [2]. However, patient compliance is often poor. Dietary strategies include modifications in fat and complex carbohydrate intake or low glycaemic index (GI) diets, all of which can be difficult to follow or maintain [3]. An alternative approach is the use of a 55 g whey preload drink which can slow gastric emptying and increases insulin, glucagon like peptide-1 (GLP1) and glucose inhibitory peptide release and lowers glucose by 3 mmol/L [4]. An alternative approach involves the use of 15-30 g glutamine which also lowers postprandial glucose by 0.3 to 1 mmol/L [5]. Fibres such as pectin [6] and guar [7] reduce gastric emptying but pectin tends to lower insulin without an effect on glucose levels in normal subjects while guar lowers both in people with diet controlled type 2 diabetes. Fat may also be used to slow gastric emptying and enhance GLP1 secretion which delays and lowers the glucose peak by 1 mmol/L but at a cost of an extra 240 kcal added to the meal [8].

We speculated that dramatically lowering the amount of whey and replacing some of the protein with guar would be just as effective at lowering postprandial glucose as 55 g of whey alone. Guar alone in dose of 4 g twice daily has been shown in one study to lower Hba1c by 0.6% suggesting that flattening of the postprandial glycemic profile can lead to clinical useful effects [9].

## Methods

### Subjects

#### Inclusion criteria

Confirmed prediabetes or type 2 diabetes from fasting glucose or oral glucose tolerance test performed by general practitioner

Fasting glucose less than or equal to 10

HbA1c less than 8.5%

Diet or metformin only

No restriction on age or BMI

#### Exclusion criteria

Attempting to lose weight or restrict caloric intake

Use of insulin or any other medication except metformin

Twenty-four subjects with pre-diabetes or moderately well -controlled type 2 diabetes were recruited by newspaper advertisement between July and December 2013. These participants consisted of 13 people with type 2 DM and 11 with prediabetes. One subject had a fasting blood glucose of 10 mmol/L while all the rest were under 9 mmol/L. Ethics approval was from the Human Ethics committee of the Alfred Hospital (number 416/12) and all volunteers gave written informed consent. The trial was registered with the Australian New Zealand Clinical Trials Registry number ACTRN12612001251819.

### Preload

The preload contained 17 g whey protein plus 3 g lactose and 5 g guar, and 1 g flavour material (including non-caloric sweetener either stevia or sucralose) dissolved in 150 ml cold water or 150 ml cold water alone.

### Protocol

The study was an unblinded 5-day continuous glucose monitoring (CGMS) study and an acute post meal fingerprick blood glucose study. Four finger prick tests were also conducted per day for optimal performance of the monitor. Six visits to the clinic were required with the first day for insertion and stabilisation of the monitor and the next 4 days for meal/drink tests. A packaged pre-test meal was provided the night before each test to standardise conditions. All morning medications were taken at least 15 minutes before the test drink.

### Test meal

The breakfast test meal consisted of 2 slices of bread, margarine and jam (3 slices for men) plus tea/coffee if desired, with a test drink 15 minutes beforehand. Tea and coffee intake for each individual was standardised to be consistent across all 4 visits. This test drink consisted of 150 ml of cold water mixed with 17 g of protein plus 5 g of fibre (the active drink) or 150 ml of plain cold water (control). The order of these two pre-meal drinks was selected at random. The active drink was taken on two of these days, and water on the other two days.

### Glucose assessment

The iPRO2 blinded CGMS (Medtronics Australasia) was inserted into the abdominal skin on a Monday afternoon. Once the signal was stable an initial fingerprick blood glucose was taken and the volunteer then went home with a prepackaged evening meal. All fingerprick glucose values were recorded on a paper log with the time of recording and returned to the clinic every 24 hours. CGMS values were taken every 5 minutes for 5 days but this analysis was confined to 3 hours after the preload to match the fingerprick data. Values from both tests and control days were averaged.

### Fingerprick test

Fingerpricks were taken before the breakfast and then every 15 min for the first hour, then at 90 min, 120 min and 180 min. Values from the two treatment and two control days were averaged.

### Statistics

The primary hypothesis was that the protein/fibre would lower the peak glucose by 1 mmol/L compared with the water and we had 99% power, p < 0.001 to show a glucose lowering of 1 mmol/L for the fingerprick tests (SD 0.6 mmol/L from a previous pilot study). Randomisation was performed using a random number generator for treatment pairs (ie treatment/control for days 1 and 2 and treatment/control for days 3 and 4). Peak change was calculated manually as the timing of the peak varied from person to person and with treatment. The whole curve over 3 hours was also analysed using repeated measures ANOVA with diabetes status and medication as between subject factors and fasting glucose as a covariate. Paired t tests were also used to examine each time point in the fingerprick tests if the overall curves were different by repeated measures ANOVA. Average glucose over 3 hours was calculated as a time weighted average of all 8 fingerprick points and all time points from the CGMS (equivalent to AUC). Data is shown as mean +/SD.

## Results

### Baseline characteristics of participants

There were 6 men and 5 women who had prediabetes, with an average age of 60 and a BMI of 32. One was treated with metformin. There were 7 men and 6 women with diabetes with an average age of 60 and a BMI of 32. Nine were treated with metformin.

### Fingerprick glucose

**Figure 1:**
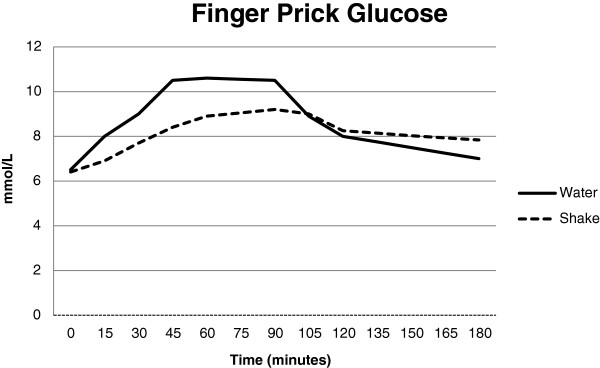
**Blood glucose over 180 min by finger prick.** Fingerpricks were taken before the test drink and then at 15, 30, 45, 60, 90, 120 and 180 minutes. The two control and two treatment test days were averaged. All time points except 120 minutes were significantly different between the curves and the repeated measures ANOVA was significantly different (Treatment p < 0.001, time p < 0.001, time by treatment p < 0.001).

The control and treatment curves were different by repeated measures ANOVA (Figure 1, Treatment p < 0.001, time p < 0.001, time by treatment p < 0.001). Blood glucose was reduced by 1.1 mmol/l at 15 min (p = 0.003), 1.3 mmol/L at 30 min (p = 0.001), 2.1 mmol/L at 45 min (p = 7 × 10^-5^) and 1.7 mmol/L at 60 minutes (p = 0.0007) and 1 mmol/L at 90 min (p = 0.02). It was higher by 0.25 mmol/L at 120 min (NS) and 0.84 mmol/L at 180 min (p = 0.003). Peak glucose was reduced by 1.4 mmol/L (p < 0.001) and the summated peak was shifted by 30 minutes. Average glucose over 3 hours was reduced by 0.77 mmol/L (p = 0.0003).

**Figure 2:**
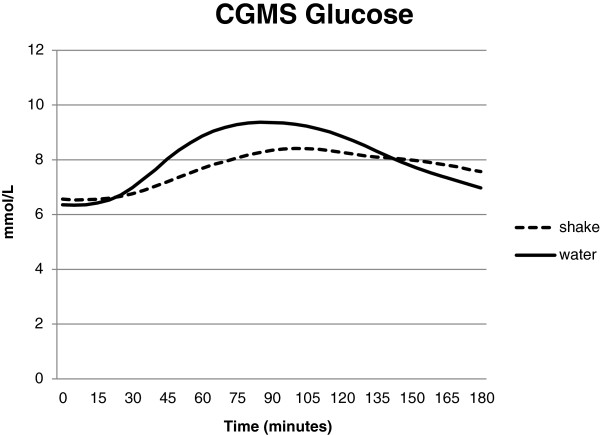
**Blood glucose over 180 min by continuous glucose monitoring.** Blood glucose values were calculated every 5 minutes. The two control and two treatment test days were averaged. The repeated measures ANOVA was significantly different (Treatment p < 0.001, time p < 0.001, time by treatment p < 0.001).

There was no difference in response between the people with type 2 DM and prediabetes and there was no relationship between fasting glucose response and the effect of the drink.CGMS: The average effect of the drink was a peak reduction of 1.04 mmol/L (p < 0.0001) with the effect ranging from +1.3 mmol/L to -2.3 mmol/L (Figure 2). The average CGMS glucose over 3 hours was lowered by 0.7 mmol/L (p < 0.001). Seven individuals showed no peak reduction and five showed no delay in peak. There was no effect beyond 3 hours. There were no adverse effects of the drink.

## Discussion

The premeal protein/fibre drink clearly lowers the peak and average glucose over three hours and delays the peak as well, demonstrating it could be a useful therapy to add to patients with prediabetes or diabetes not optimally controlled by diet or diet plus metformin. The average lowering of nearly 1 mmol/l over 3 hours with a peak reduction of 2 mmol/L is exactly equivalent to [10] or greater than [11] the effect of sitagliptin on acute glucose profile. Sitagliptin can lower HbA1c by an average of 0.7% compared with placebo [12]. This finding with sitagliptin matches the findings with dietary carbohydrate where in 901 free living patients with type 2 diabetes those in the lowest quintile of glycemic index and glycemic load had an HbA1c 1% lower than those in the highest quintile and the difference in post meal glucose at one hour between these groups was about 2 mmol/L [13]. This premeal protein/fibre drink treatment would appear to outperform low GI meal planning but long term data is not yet available so we do not know how well patients will comply with a pre-meal drink regimen long term. In the Jenkins low GI- 6 month study in type 2 diabetes [14], Hba1c was lowered by 0.32%. The difference between the diets was 13 GI units. A meta-analysis of 16 studies using GI in meal planning showed an average reduction in HbA1c of 0.27% (p = 0.03) and fructosamine of 0.1 mmol/L (p = 0.05) [15].

Whey drinks fed 30 minutes before an ad libitum pizza in young healthy men show a clear dose response from 10-40 g of whey with a peak reduction of 1.8 mmol/L with 20 g whey at 30 minutes after the meal. Thirty g of whey was similar but 40 g of whey reduced glucose by 2.4 mmol/L at 35 minutes [16]. For both 20 g and 40 g of whey food intake and thus carbohydrate intake was reduced by 13-30% accounting for some of the glucose reduction. Glucose reduction with 17 g of whey was greater in the current study and although not formally tested the addition of guar to whey would appear to add to its glucose lowering effect. In another study in 14 healthy, lean subjects 9 g of whey protein isolate taken immediately before a fixed meal lowered glucose at 45 min by 2.6 mmol/L and elevated insulin and GLP1 [17].

The mechanism of the effect on postprandial glucose would appear to be via slowing gastric emptying from both guar [7] and protein [4] as well as via protein induced insulin release [4, 5]. With 55 g of whey Ma et al. [4] demonstrated a 3 fold increase in insulin at 90 minutes while Samoch-Bonetet al. [5] showed that 15 g of glutamine increased insulin from 450 to 600 pmol/L and delayed the insulin peak from 30 to 60 minutes and its effect was more marked than 100 mg of sitagliptin. Guar alone in a dose of 4 g twice daily has been shown in one study to lower Hba1c by 0.6% suggesting this degree of flattening of the postprandial glycemic profile with guar alone can lead to long term glucose lowering and clinically useful effects [9]. Thus with the treatment described in this paper we would anticipate an equal contribution from protein and guar to glucose lowering although we did not set out to specifically elucidate the separate and combined effects of whey and guar but only tested a practical combination drink. GLP1 and PYY are elevated by whey more than by glucose preloads before a pizza meal [18] suggesting GLP1 may be important in the effect of the drink.

In terms of cardiovascular complications a 1 hour postprandial glucose level predicts myocardial infarction and death in people with newly diagnosed with type 2 diabetes [19]. In both men and women with diabetes who had myocardial infarctions a post lunch difference of about 2 mmol/L (time not specified) was seen compared with people without disease [20]. In a meta- analysis of 7 acarbose studies (an alpha-glucosidase inhibitor which reduces glucose absorption) 1 hour postprandial glucose was reduced by 1.6 mmol/L and 2 h glucose by 2.2 mmol/l while HbA1c was reduced by 0.6% and myocardial infarctions were reduced by 64%. Acarbose also reduced weight, triglyceride and systolic blood pressure [21]. Thus in this study the effect on 1 hour glucose is equivalent to acarbose but the drink is unlikely to have an effect on triglyceride and blood pressure but it may reduce weight long term. Although acarbose causes abdominal discomfort because of colonic fermentation of carbohydrate this did not occur in this study as we believe carbohydrate absorption was slowed but not inhibited to any degree. We are currently performing a 6 month randomised controlled trial examining the effect of a protein/fibre pre meal drink on HbA1c, weight and blood lipids.

In conclusion we have demonstrated that a low calorie protein/fibre drink acutely lowers glucose by 2 mmol/L in people with prediabetes and type 2 diabetes and thus could be a potential long term dietary strategy to reduce the glycemic impact of high carbohydrate meals. The drink is palatable and could be used on a daily or twice daily basis but we have no data on long term compliance yet.
